# The effectiveness of allied health and nurse practitioner models-of-care in managing musculoskeletal conditions in the emergency department: a systematic review and meta-analysis

**DOI:** 10.1186/s12873-023-00925-4

**Published:** 2024-01-17

**Authors:** Simon P Vella, Alla Melman, Danielle Coombs, Christopher G Maher, Michael S Swain, Elizabeth Monk, Gustavo C Machado

**Affiliations:** 1 School of Public Health, Faculty of Medicine and Health, The University of Sydney, Sydney, Australia; 2 Sydney Musculoskeletal Health and Institute for Musculoskeletal Health, Sydney Local Health District, Sydney, Australia; 3 Royal Prince Alfred Hospital, Level 10N, King George V Building, Missenden Road, Camperdown, NSW 2050 Australia; 4 Department of Chiropractic, Faculty of Medicine, Health and Human Sciences, Macquarie University, Sydney, Australia; 5 St George Hospital Emergency Department, Sydney, Australia

**Keywords:** Health service, Emergency department, Allied health, Nursing, Musculoskeletal pain, Effectiveness

## Abstract

**Background:**

Musculoskeletal conditions are the most common health condition seen in emergency departments. Hence, the most effective approaches to managing these conditions is of interest. This systematic review aimed to evaluate the effectiveness of allied health and nursing models of care for the management of musculoskeletal pain in ED.

**Methods:**

MEDLINE, EMBASE, CINAHL and LILACS databases were searched from inception to March 2023 for published randomised trials that compared the effectiveness of allied health and nursing models of care for musculoskeletal conditions in ED to usual ED care. Trials were eligible if they enrolled participants presenting to ED with a musculoskeletal condition including low back pain, neck pain, upper or lower limb pain and any soft tissue injury. Trials that included patients with serious pathology (e.g. malignancy, infection or cauda equina syndrome) were excluded. The primary outcome was patient-flow; other outcomes included pain intensity, disability, hospital admission and re-presentation rates, patient satisfaction, medication prescription and adverse events. Two reviewers performed search screening, data extraction, quality and certainty of evidence assessments.

**Results:**

We identified 1746 records and included 5 randomised trials (n = 1512 patients). Only one trial (n = 260) reported on patient-flow. The study provides very-low certainty evidence that a greater proportion of patients were seen within 20 min when seen by a physician (98%) than when seen by a nurse (86%) or physiotherapist (77%). There was no difference in pain intensity and disability between patients managed by ED physicians and those managed by physiotherapists. Evidence was limited regarding patient satisfaction, inpatient admission and ED re-presentation rates, medication prescription and adverse events. The certainty of evidence for secondary outcomes ranged from very-low to low, but generally did not suggest a benefit of one model over another.

**Conclusion:**

There is limited research to judge the effectiveness of allied health and nursing models of care for the management of musculoskeletal conditions in ED. Currently, it is unclear as to whether allied health and nurse practitioners are more effective than ED physicians at managing musculoskeletal conditions in ED. Further high-quality trials investigating the impact of models of care on service and health outcomes are needed.

**Supplementary Information:**

The online version contains supplementary material available at 10.1186/s12873-023-00925-4.

## Introduction

Musculoskeletal conditions are the leading cause of years lived with disability globally and contribute to approximately 5–7% of all emergency department (ED) presentations [1–3]. These presentations are also costly to health systems [4–6]. In the United States, it is estimated that US$380 billion is spent on healthcare for musculoskeletal conditions annually [4]. In Australia, musculoskeletal conditions cost the health system AUD$16.6 billion per year with two-thirds of this expenditure occurring in hospitals [5, 6]. Nurse practitioners and primary-contact allied health clinicians such as physiotherapists can assess and treat most patients with simple musculoskeletal conditions without requiring medical staff referral in primary care and in some ED settings [7, 8]. These models of care may be an effective strategy to improve patient-flow and satisfaction with care for patients presenting to the emergency department with musculoskeletal conditions [9–11], and may reduce pressure on emergency physicians who are required to distribute their time to ensure each presenting patient receives adequate care regardless of their complaint [12].

There is evidence to suggest that ED physiotherapists managing musculoskeletal conditions enhance patient-flow by reducing ED waiting and treatment times [13–18]. For instance, an observational study with 524 patients in the United States showed that secondary-contact physiotherapists (i.e. patients were referred to physiotherapy by a doctor) managing musculoskeletal conditions had shorter ED length of stay of 4-hours (range 1-26-hours) compared to patients managed by an ED physician (6.2-hours; range 1-28-hours) [18]. One observational study in Australia with 1060 patients showed that primary-contact physiotherapists (i.e. patients receive direct physiotherapy management without doctor referral) managing musculoskeletal conditions reduced the time from triage to being seen in ED by 10-minutes (SD 11-minutes) and reduced overall ED length of stay by 108-minutes (SD 88-minutes) when compared to patients who received routine care from an ED physician [13]. Additionally, one observational study has shown that patients managed by primary-contact physiotherapists compared to an ED physician or nurse practitioner have less pain (1-point versus 4-points on a 0–10 pain scale) and lower levels of disability measured using the Neck Disability Index and the Modified Oswestry Low Back Pain Disability questionnaire (9% disability versus 33% disability) after treatment [19]. Furthermore, patients managed by primary-contact physiotherapists were discharged from ED sooner (i.e. within 4 h, 93% versus 75%), and received less imaging (30% versus 43%) and less opioids (18% versus 33%) in observational studies [15, 18–20].

Evidence from observational studies also support nurse practitioner models of care in the management of musculoskeletal conditions in the ED [9, 16, 21]. For example, one observational study with 320 patients in Australia found that two-thirds of patients managed by nurse practitioners rated their satisfaction with care as ‘excellent’ compared to 50% of patients who were managed by an ED physician [16]. Patients managed by nurse practitioners for traumatic and soft tissue injuries also had shorter wait times [9, 21]. That is, patients managed by a nurse practitioner were seen within 12-minutes (IQR 6-28-minutes), compared to patients managed by a physician (31-minutes, IQR 12-76-minutes) [21]. Additionally, patients managed by nurse practitioners were discharged from ED sooner than those managed by a physician (94-minutes versus 170-minutes) [21]. While observational research supports the use of nursing and allied health models of care for the management of musculoskeletal conditions in ED, these studies are at inherent risk of selection bias and the evidence of effectiveness from randomised trials remains unclear.

Previous systematic reviews have attempted to evaluate the effectiveness of physiotherapists [22] and nurse practitioners [23] on ED efficiency and quality of care. However, these two reviews are not focussed on musculoskeletal conditions, combine observational and experimental study designs, do not align recommendations with the level of certainty of the evidence (using the GRADE approach), and only included articles of English and French language. No research has yet comprehensively appraised and synthesised the research on effectiveness of nurse and allied health practitioners managing musculoskeletal conditions in ED. Understanding the effectiveness of these models of care is vital to reducing the burden that these conditions place on ED. The aim of this review is to evaluate the effectiveness of nurse practitioners and allied health practitioners managing musculoskeletal conditions in ED.

## Methods

### Study design

This systematic review and meta-analysis was prospectively registered with Open Science Framework (10.31219/osf.io/mzt49) and was written in accordance with the Preferred Reporting for Systematic Reviews and Meta-analyses (PRISMA 2020) statement [24].

### Search strategy

Electronic databases (MEDLINE, EMBASE, CINAHL and LILACS) were searched from inception to March 2023. Relevant search terms and their variations were used to construct a search strategy for each database (Appendix 1). There was no language restriction for articles. Search results were exported to EndNote 20 (Clarivate, Philadelphia, US) where duplicates were removed and then imported to Covidence (Veritas Health Innovation, Melbourne, Australia) for screening. Two reviewers (SPV and AM) independently performed title and abstract screening and removed clearly irrelevant articles. The same two reviewers independently screened full text of potentially eligible articles to determine inclusion. One reviewer (SPV) performed backward-citation tracking on included articles to identify trials that were not retrieved using the described search strategy. Disagreements regarding inclusion were resolved in consultation with a third reviewer (GCM).

### Study selection

We included randomised controlled trials that compared the effectiveness of care delivered by ED nurse or allied health practitioners (either primary-contact or secondary referral) against usual ED medical care in the management of musculoskeletal conditions in ED. Nurse practitioners have additional training in clinical roles such as ordering investigations, diagnosis, management, prescribing and patient discharge planning. Allied health practitioners providing care in the ED include physiotherapists, chiropractors and occupational therapists. Trials were required to compare models of care provided by a nurse practitioner and/or an allied health practitioner to usual medical care (i.e. where a doctor primarily manages the patient within ED). Trials were eligible if they enrolled participants presenting to ED with musculoskeletal pain including low back pain, neck pain, upper or lower limb pain and any soft tissue injury. Trials that included patients with serious pathology (e.g. malignancy, infection or cauda equina syndrome) were excluded. There was no restriction applied to age, duration or region of musculoskeletal pain. Studies were required to be published in a peer-reviewed journal. There were no language restrictions applied to the eligibility criteria.

### Outcomes

The primary outcome of this systematic review was patient-flow, and includes measures such as wait time (time to be seen by a health professional), duration of clinical care (time a health professional spent treating each participant), and overall ED length of stay (time between patient arriving in the ED to when the patient physically departs the ED).

Secondary outcome measures included pain intensity, disability, hospital admission and ED re-presentation rates, patient satisfaction with care, analgesic medication prescription or administration, and adverse events. Measures for pain intensity were converted to a common 0-100 scale whereby 0 relates to no pain and 100 denotes worst possible pain. Converting scores to a 0-100 scale has been performed in previous systematic reviews evaluating pain outcomes [25–27], and aligns with expressing minimum important clinical differences as points on a 0-100 scale [28–30]. Disability measures included, for example, the Roland Morris Disability Questionnaire (RMD), Disability of the Arm, Shoulder and Hand (DASH) and the Lower Extremity Functional Scale (LEFS), and due to the broad range of tools, disability scores were left unconverted. Hospital admission and ED re-presentation rates included the proportion of participants who were admitted from ED to an inpatient unit or re-presented to ED within 30 days of their index presentation with a similar musculoskeletal condition. Patient satisfaction represented the level of satisfaction the patient reported toward the care they received in ED, measured via questionnaires. Medication prescription or administration and adverse events included the proportion of participants who received medication prescription at ED discharge or were given a medication during the ED stay, or experienced adverse events (as defined by study authors) during the ED stay.

Follow-up time points were defined as immediate-term (measured during the ED stay), short-term (measured after ED discharge but before 3-months), intermediate-term (measured between 3 and 12-months after ED discharge) and long-term (measured > 12-months after ED discharge).

### Data extraction

One reviewer (SPV) extracted data into a specifically designed Excel spreadsheet (Microsoft Corporation, US) and a second reviewer (AM) independently verified the extracted data. Disagreements relating to extracted data were resolved by arbitration with a third reviewer (GCM). Extracted data included study characteristics (e.g. author, country, year, and type of health profession), sample characteristics (e.g. sample size, age, diagnosis, type of care for example education, exercise prescription, mobility aids, and advice on over the counter medication, duration of symptoms, intervention and comparator) and outcome data (e.g. time spent in ED, pain and disability scores, hospital admission and re-presentation rates, patient satisfaction scores, proportion of patients receiving analgesic medication in ED or a prescription at discharge, number and proportion of patients reporting adverse events) at each follow-up time point. Where possible, for each continuous outcome we extracted post intervention means, standard deviations (or 95% confidence intervals), and number of participants in each group. For dichotomous outcomes, we extracted the number of participants that experienced an event (e.g. hospital admission), no event (e.g. no hospital admission) and the total number of participants in each group. We contacted authors of the included studies requesting data if outcome data were missing, incomplete, or if we were unsure about the data presented.

### Risk of bias and certainty of evidence

Two reviewers (SPV and AM) rated risk of bias of trials using the Physiotherapy Evidence Database (PEDro) tool [31] and assessed the certainty of evidence using the Grading Recommendations, Assessment, Development and Evaluation (GRADE) approach [32]. The PEDro scale is a valid and reliable tool to assess the internal validity of trials [33]. One point is awarded when each criterion (except for the first item– eligibility criteria) is satisfied and the total numerical score (maximum score up to 10) determines the trials overall methodological quality.

Certainty of evidence reflects our confidence that the estimates of effect are correct [34]. Certainty of evidence was downgraded by one level if serious issues relating to study design bias, inconsistency (i.e. heterogeneity), imprecision and small study effects (Appendix 2– GRADE approach). We did not grade indirectness as study populations, interventions and comparators were similar across studies. Heterogeneity for pooled analyses was determined using the I^2^ statistic in conjunction with the tau (T) statistic to describe between-study variance [35]. Inconsistency was not assessed when findings were based in single studies. Certainty of evidence was rated as very-low, low, moderate, and high. Very-low certainty suggests that the true effect may be markedly different from the estimate effect (i.e. low confidence) and high certainty is defined as high confidence that the true effect is similar to the estimate effect. Disagreements in risk of bias and certainty of evidence assessments were resolved by consensus.

### Data synthesis and analysis

Descriptive statistics were used to summarise study characteristics. We planned to analyse the primary outcome ‘patient-flow’ (in minutes) as a continuous outcome. Secondary outcomes such as pain intensity and disability scores were converted to a common 0-100 scale (0 no pain or disability, 100 maximum pain or disability). Continuous outcome measures such as patient-flow, pain intensity and disability were reported as means (SD, or 95% confidence interval). If a study did not report standard deviations, we used estimation methods recommended by the Cochrane Handbook for Systematic Reviews of Intervention [36], and followed the formulae reported under Sect. 6.5.2.2. to obtain the standard deviations for group means. Dichotomous outcomes such as hospital admission and ED re-presentation rates, and adverse events were reported as proportions (95% confidence intervals). If categorical data (e.g. mild, moderate, and severe) were presented in individual trials we contacted the study authors to request summaries of continuous data, otherwise data were excluded from our analysis. If trials were clinically homogenous, we pooled trial results using random-effects meta-analysis using Comprehensive Meta-analysis, V4 (BioStats, Inc). For pain intensity, pooled mean differences below 10-points were considered clinically unimportant [37], and to account for variation in reporting of disability measures we report the standardised mean difference (SMD) in units of standard deviations to describe the overall intervention effect [36]. If clinical heterogeneity existed between study interventions and outcomes that impaired our ability to perform meta-analysis we narratively described study results. We intended to conduct subgroup analyses to investigate differences in outcomes between the health professions.

## Results

### Study selection

**Fig. 1:**
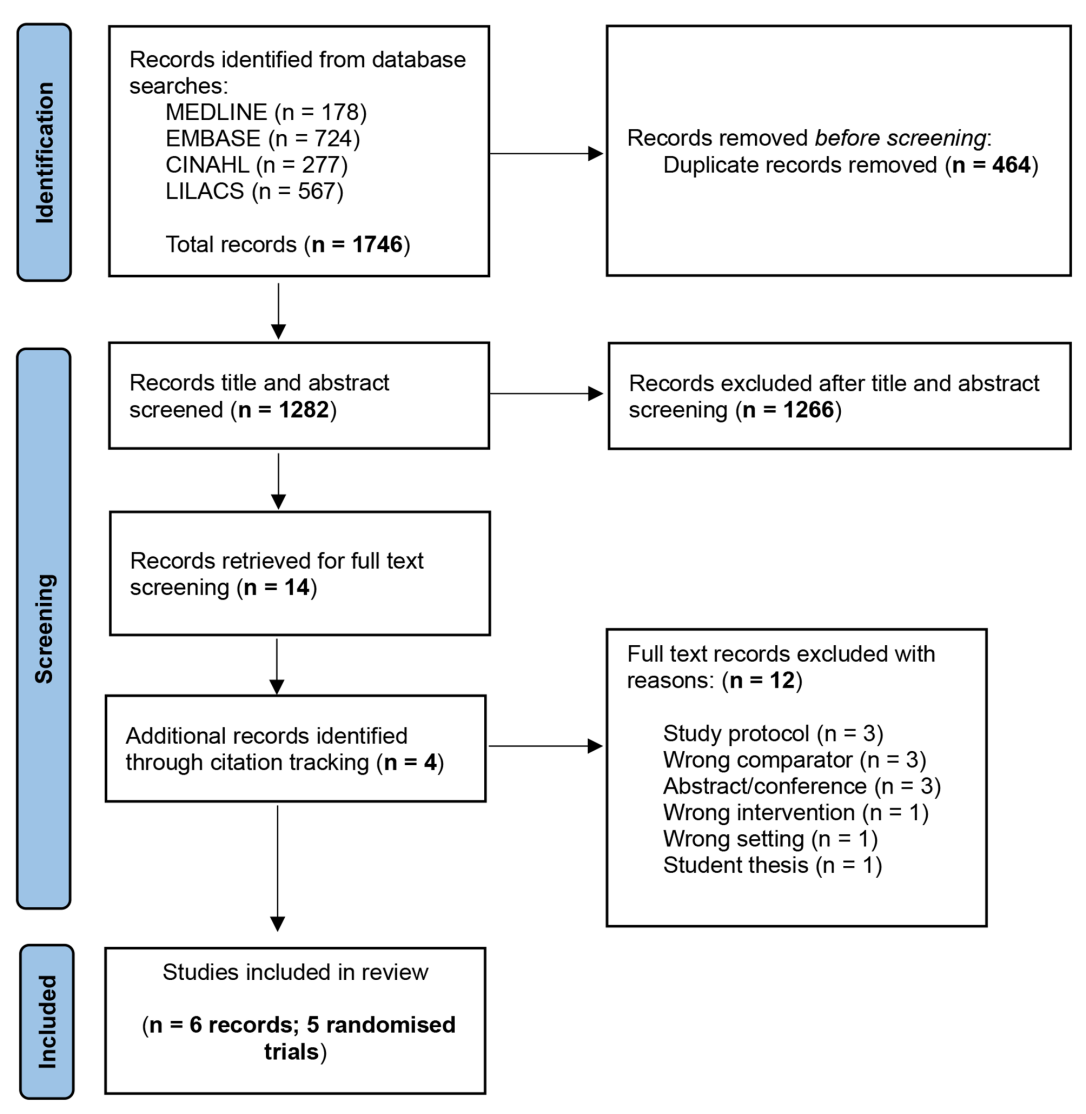
Search flow chart with reasons for exclusion

The search returned 1746 results and after duplicates (n = 464) were removed, 1282 articles were imported to Covidence for title and abstract screening. Fourteen full-text articles were reviewed, and four additional articles were identified through backward-citation tracking. We included six records reporting five randomised controlled trials [17, 20, 38–41] in our systematic review. Two records [39, 40] used data from the same trial but report on different outcomes (i.e. patient outcomes and costs). Search results and reasons for exclusion are presented in Fig. 1.

### Study characteristics

Five trials included data for 1512 participants presenting to ED with musculoskeletal pain. Four trials (five records) [17, 38–41] included patients with soft tissue injuries and one trial [20] included patients with acute low back pain. Trials were from the United Kingdom [39–41] Canada [17], Hong Kong [20] and Australia [38]. The sample size of included trials ranged from 78 to 766 participants with a mean age that ranged from 32.9 to 70.1 years. Three trials investigated primary-contact physiotherapy care [17, 20, 41], one trial assessed primary-contact physiotherapy and nurse practitioner care [39, 40], and one trial evaluated secondary referral to physiotherapists [38]. Included trials compared care delivered by physiotherapy or nurse practitioners to care provided primarily by an ED physician. Study characteristics are presented in Table 1.

**Table 1:** Characteristics of included studies

Author, year	Country	Study setting	Sample size	Age, mean (SD)	Diagnosis	Duration of symptoms	Study treatment	Outcome	Follow-up time points
Gagnon, 2021 [14]	Canada	Centre Hospitalier de l’Université Laval ED	Int: n = 40 (M:18) Con: n = 38 (M:26)	Int: 36.6 (17.3) Con: 44.1 (17.3)	Minor musculoskeletal disorder (i.e. categorised as triage score 3, 4 or 5 according to the Canadian Emergency Department Triage and Acuity Scale	Ranged from 0 to greater than 12 weeks	Primary care physiotherapy vs. usual care (at discretion of ED physician)	Pain intensity (0–10) Disability (0–10) Inpatient admission Re-presentation rate Medication prescription Adverse events	Post-intervention (at ED), 1-month and 3-month
Lau, 2008 [17]	Hong Kong	ED of local hospital	Int: n = 55 (M:21) Con: n = 55 (M:22)	Int: 52 (18) Con: 49 (15)	Acute low back pain (with or without referred leg pain)	Within 24 h of ED admission	Primary care physiotherapy vs. usual care	Pain intensity (0–10) Disability (0–24)	Admission to ED, Discharge from ED, admission to physiotherapy outpatient clinic, 1-month, 3-month, 6-month
Richardson, 2005 [38]	United Kingdom	ED of district hospital	Int: n = 382 (M:232) Con: n = 384 (M:232)	Int: 38.7 (16.1) Con: 40 (16.4)	musculoskeletal disorders categorised as triage score 3, 4 or 5 according to United Kingdom emergency triage system	Ranged from 0 to greater than 48 h	Primary care physiotherapy vs. usual care (i.e. routine ED care)	Time to return to activities Patient satisfaction	3-month and 6-month
Jesudason, 2011 [35]	Australia	Royal Adelaide hospital	Int: n = 93 (M:34) Con: n = 93 (M:29)	Int: 72 (19.0) Con: 68.1 (15.5)	Patients deemed to require physiotherapy by ED physician or nurse: musculoskeletal disorder (n = 117), other conditions (n = 71)	Not reported	Secondary referral to physiotherapy vs. usual care	Inpatient admission Re-presentation rate Patient satisfaction Adverse events	1-month
McClellan, 2012 [36] and 2013 [37]	United Kingdom	ED of University Hospitals Bristol	Int 1: n = 84 (M:50) Int 2: n = 83 (M:39) Con: n = 93 (M:57)	NR	Peripheral soft tissue injury (with no associated bone fracture, ongoing prior injury or systemic disease/disorder).	Less than 72 h	Primary care physiotherapy or Nurse practitioner vs. usual care	Treatment time Disability Medication prescription	2-weeks and 8-weeks

Int; intervention group, Con; control group, M; male, ED; emergency department, Int 1; physiotherapy group, Int 2; Emergency nurse group, NR; Not reported

### Risk of bias in trials

Table 2 reports risk of bias assessments of the included trials using the PEDro scale. Three trials were graded as low risk of bias [20, 38, 39] and two trials were graded as high risk of bias with a PEDro score < 7 [17, 41]. Each trial provided appropriate random allocation, between-group comparisons and point estimates with measures of variance. All included trials failed to blind subjects and therapists. Two trials failed to blind assessors [17, 41], provide adequate follow-up [17, 41], and to provide baseline between-group comparisons [38, 39], and one trial did not perform intention-to-treat analysis [17].

**Table 2:** Risk of bias of included studies according to the PEDro scale

Author/Year	Eligibility Criteria*	Random allocation	Concealed allocation	Baseline comparability	Blind subjects	Blind therapists	Blind assessors	Adequate follow-up	Intention-to-treat analysis	Between-group comparisons	Point estimates and variability	Total
McClellan (2012)	Yes	Yes	Yes	No	No	No	Yes	Yes	Yes	Yes	Yes	**7**
Jesudason (2012)	Yes	Yes	Yes	No	No	No	Yes	Yes	Yes	Yes	Yes	**7**
Gagnon (2021)	Yes	Yes	Yes	Yes	No	No	No	No	No	Yes	Yes	**5**
Lau (2008)	Yes	Yes	Yes	Yes	No	No	Yes	Yes	Yes	Yes	Yes	**8**
Richardson (2005)	Yes	Yes	Yes	Yes	No	No	No	No	Yes	Yes	Yes	**6**

*Eligibility criteria item does not contribute to total score

### Certainty of evidence: GRADE assessment

The overall quality of evidence for physiotherapy and nurse practitioner care on primary and secondary outcomes ranged from very-low to low (Table 3 and supplementary material). We did not judge certainty of evidence for adverse events due to studies providing little to no data. Additionally, we were unable to assess for small study-effects due to few studies included. For pain at ED discharge, 1-month and 3-months, variance between study effects (T^2^) was 65.2, 82.6 and 117.9 and the I^2^ statistic was 58.0%, 73.4% and 81.6%, respectively. For disability at 1-month and 3-months, T^2^ for both time-points was 0.0 and the I^2^ statistic was 18.1% and 5.3%, respectively.

**Table 3:** Summary of findings and certainty of evidence for included study outcomes

	Summary of findings		Certainty of evidence			
	No of participants (No of RCTs)	Effect size (95% CI)	Study design	Inconsistency	Imprecision	Certainty of evidence
**Patient-flow**						
Proportion of patients that had contact-time with clinicians of 20 min or less	260 (1)	Physician: 98% of patients Nurse: 86% of patients Physiotherapist: 77% of patients	Downgraded	Not assessed^#^	Downgraded	Very-low^1^
**Pain (0-100)**						
ED-discharge	155 (2)	MD -10.9 (-24.8 to 2.9)	Downgraded	Downgraded	Downgraded	Very-low
1-month	163 (2)	MD -10.5 (-25.0 to 4.1)	Downgraded	Downgraded	Downgraded	Very-low
3-months	161 (2)	MD -8.1 (-24.7 to 8.6)	Downgraded	Downgraded	Downgraded	Very-low
**Disability**						
1-month	386 (3)	SMD − 0.2 (-0.4 to 0.0)	Downgraded	Unchanged	Downgraded	Low
3-months	374 (3)	SMD − 0.2 (-0.4 to 0.0)	Downgraded	Unchanged	Downgraded	Low
**Inpatient admission**						
Within 1-month	186 (1)	Difference in proportions 4% (-18 to 9%)	Unchanged	Not assessed^#^	Downgraded	Very-low
**Re-presentation rate**						
Within 1-month	186 (1)	Difference in proportions 3.5% (-6 to 13%)	Unchanged		Downgraded	Very-low
**Medication prescription**						
At ED-visit	260 (1)	Physician: 42% of patients, Nurse: 23% of patients, Physiotherapist: 4% of patients	Downgraded		Downgraded	Very-low^2^
	76 (1)	Difference in proportions − 24% (-46% to -2%)	Downgraded		Downgraded	Very-low
1-month	76 (1)	Difference in proportions − 40% (-62% to -18%)	Downgraded		Downgraded	Very-low
3-months	62 (1)	Difference in proportions − 11% (-29 to 9%)	Downgraded		Downgraded	Very-low
**Recommended use of medications (over-the-counter medications)**						
At ED-visit	76 (1)	Difference in proportions 59% (41 to 76%)	Downgraded		Downgraded	Very-low
1-month	63 (1)	Difference in proportions -19% (-42 to 2%)	Downgraded		Downgraded	Very-low
3-months	62 (1)	Difference in proportions -25% (-40% to -2%)	Downgraded		Downgraded	Very-low

Key: RCTs; randomised controlled trials, MD; mean difference, SMD; standardised mean difference

Note: Unable to assess small study effects due to few studies included

^#^Inconsistency was not assessed as findings were based in single studies or heterogeneity existed between measurement of outcome

^1^Study provided categorical data regarding time patients spent with healthcare professional, ^2^Study provided no confidence intervals around estimates

### Primary outcome

Only one trial (n = 260) reported on patient-flow [39]. The trial evaluated the amount of contact-time spent with patients between physiotherapists, nurse practitioners and emergency physicians. The trial provides very-low certainty evidence that a greater proportion of patients had contact-time of 20-minutes or less when primarily managed by a physician (98%) than when managed by a nurse (86%) or physiotherapist (77%).

### Secondary outcomes

#### Pain intensity

Two trials (n = 163 patients) provided very-low certainty of no effect on pain intensity at ED discharge, 1-month and 3-month follow-up between physiotherapist and physician care (Table 3) [17, 20]. The pooled mean difference in pain intensity at ED discharge was − 10.9 (95% CI -24.8 to 2.9), at 1-month was − 10.5 (95% CI -25.0 to 4.1), and at 3-months follow-up was − 8.1 (95% CI -24.7 to 8.6). Only one trial reported pain intensity at 6-month follow-up and found no difference between groups (mean difference 0.0; 97.5% CI -9.0 to 8.0) [20].

### Disability

Three trials (n = 386 patients) provided low certainty evidence of no difference in disability outcomes at 1-month and 3-month follow-up between physiotherapists, nurse practitioners and physicians (Table 3) [17, 20, 39]. There was no difference in disability reported between patients managed by physiotherapists, nurse practitioners or ED physicians at 1-month (SMD − 0.2, 95% CI -0.4 to 0.0) and 3-months (SMD − 0.2, 95% CI -0.4 to 0.0).

### Hospital admission and ED re-presentation

Two trials (n = 262 patients) reported on inpatient admission and hospital re-presentation but presented categorical and continuous data that could not be pooled [17, 38]. The Canadian trial (n = 76) included rare events (and zero counts) for the physiotherapy and control group at each follow-up time point and due to the small sample size did not contribute to the certainty of evidence for inpatient admission and ED re-presentation [17].

The Australian trial (n = 186 patients) provided very-low quality evidence of no difference in inpatient admissions between physiotherapists and ED physicians within 1-month of ED presentation (difference in proportions 4%; 95% CI -18 to 9%) [38]. The authors reported that 29 participants (out of 93) in the physiotherapy group were admitted to hospital, compared to 33 participants (out of 93) in the control group. The Canadian trial reported no inpatient admission in the physiotherapy group (out of 40) compared to one participant (out of 36) admitted to hospital after physician care [17]. At 1-month, one participant in the physiotherapy group (out of 31) and one participant in the control group (out of 32) were admitted to hospital, and at 3-months no participants received inpatient admission.

The Australian trial provided very-low quality evidence of no difference in ED re-presentation rates within 1-month (difference in proportions 4%, 95% CI -6 to 13%) [38]. Twelve participants in the physiotherapy group (out of 91) re-presented to ED within 30 days of their initial presentation, compared to 9 participants (out of 93) in the control group [38]. The Canadian trial reported no re-presentations for musculoskeletal conditions to ED in the physiotherapy group (out of 31) compared to seven (out of 32) in the physician group at 1-month, and no participants in the physiotherapy group (out of 32) compared to one participant in the control group (out of 30) re-presented to ED within 3-months [17].

### Patient satisfaction

Two trials (n = 952) reported patient satisfaction [38, 41]. One trial provided low quality evidence suggesting that patients were more satisfied with physiotherapy assessment compared to ED physician assessment (difference in proportions 15%, 95% CI 9 to 21%) [41]. The trial used five questions scored on a 5-point Likert scale to measure patient satisfaction, though the questions that were asked and the timepoints at which patients provided their satisfaction were not reported [41]. The authors reported that 89% of patients in the physiotherapy group (n = 278) were satisfied with care compared to 74% of patients (n = 280) who received care by an ED physician or nurse practitioner. The other trial provided very-low quality evidence of no difference in patient satisfaction toward delivered care when measured between 7 and 21 days post ED presentation [38]. Patients were asked whether they were ‘overall satisfied with treatment received’ and the authors found that 82% of patients who received physiotherapy care (out of 62) were satisfied with care, compared to 87% of patients who received care from an ED physician (out of 62) [38].

### Medication prescription or recommended use of medication

Two trials (n = 338) provided very-low quality evidence suggesting that physiotherapists prescribed less medication compared to ED physicians at ED-visit and 1-month [17, 39]. The Canadian trial reported that 17 patients in the physiotherapy group (out of 40) compared to 24 patients in the usual ED care group (out of 36) were prescribed analgesic medication at ED visit, and at 1-month after discharge, 10 patients in the physiotherapy group (out of 31) versus 23 patients in the ED physician group (out of 32) were using prescription medication [17]. Additionally, the English trial reported medications administered during ED visit and included medications such as ibuprofen, paracetamol, opioids, and non-steroidal anti-inflammatories [39]. Three patients in the physiotherapy group (out of 84) were administered medication during ED visit compared to 19 patients in the nurse group (out of 83) and 38 patients in the ED physician group (out of 93) [39]. The Canadian trial provided very-low quality evidence of no difference in analgesic medication prescription at 3-months after discharge [15]. Seven patients in the physiotherapy group (out of 32) were prescribed analgesic medications versus 10 patients in the usual ED care group (out of 30) at 3-months after discharge.

The Canadian trial provided very-low quality evidence suggesting that physiotherapists recommended the use of over-the-counter medications more than ED physicians at ED visit, but there was very-low quality evidence of no difference at 1-month after discharge. Conversely, at 3-months after discharge, the same trial provided very-low quality evidence suggesting that ED physicians recommended the use of over-the-counter medications more than physiotherapists.

The authors did not provide a definition of what over-the-counter medications were [15]. Twenty-eight patients in the physiotherapy group (out of 40) were recommended to use over-the-counter medications compared to 4 patients in the ED physician group (out of 36) during ED-visit [15]. At 1-month, twelve patients in the physiotherapy group (n = 31) used over-the-counter medications compared to 18 patients in the ED physician group (n = 32), and at 3-months after discharge 6 patients in the physiotherapy group (out of 32) used over-the-counter medications compared to 13 patients in the usual ED care group (out of 30) [17].

### Adverse events

Two trials (n = 264) reported no adverse events [17, 38]. One trial used a self-reported online questionnaire to collect adverse event data at 1 and 3-months [17]. The other trial provided no details of how adverse event data were collected [38]. We did not judge the certainty of evidence for adverse events due to little to no available data.

## Discussion

### Principal findings

Our review identified five trials (six records) that investigated nurse practitioner and physiotherapy management for musculoskeletal conditions in ED. One trial provided very-low certainty of evidence that no model of care (i.e. physiotherapy versus nursing versus emergency physician models of care) was superior than another with regard to patient-flow [39]. There was no difference in pain intensity (very-low certainty evidence) and disability (low certainty evidence) between patients managed by ED physicians and those managed by physiotherapists. There was limited to no evidence on patient satisfaction, inpatient admission and ED re-presentation rates, medication prescription, and adverse events. The overall quality of evidence for these outcomes (excluding adverse events) ranged from very-low to low.

It is important to interpret the findings of our review with caution as the evidence comes from small trials and GRADE indicates very-low and low certainty [17, 20]. Our findings highlight the uncertainty surrounding the effectiveness of physiotherapy management for musculoskeletal conditions in ED and identifies a paucity of evidence toward patient-flow outcomes. While the findings of our meta-analysis show less pain intensity in the physiotherapy group compared to usual ED medical care, there was variation in effect-estimates. For example, pain intensity (scored on a 0-100 scale) in the physiotherapy group ranged from a reduction in pain of 25-points to an increase in pain of 9-points at 3-months following ED discharge, when compared to usual ED care. Furthermore, the pooled mean differences for pain intensity at ED discharge (-10.9), 1-month (-10.5), and 3-months (-8.1) following ED discharge were likely too small to be of clinical importance. Similarly, there were no significant effects on disability between patients managed by physiotherapists and ED physicians. Despite synthesising the available data from randomised controlled trials to evaluate physiotherapy and nurse models of care, the effectiveness of these models for the management of musculoskeletal conditions in ED remains unclear.

Our review expands on previous systematic reviews that have evaluated physiotherapy and nursing models of care for the management of musculoskeletal conditions in ED [22, 23, 42]. A previous review evaluating the benefits of physiotherapy models of care for musculoskeletal conditions in ED included 15 studies (10 observational studies, three randomised trials and two experimental studies) [22]. The authors concluded that evidence supported the use physiotherapy management in ED in terms of efficacy, safety and access of care, and patient satisfaction. The review included three of the trials that we included in our review [20, 39–41]. The authors narratively described pain and disability outcomes and suggested that physiotherapists were more effective for reducing pain and were as effective as usual ED medical care in terms of disability. Our review included meta-analyses of pain and disability outcomes and has shown that uncertainty surrounding the effectiveness of physiotherapy models of care still exists. Another review evaluated the impact of nursing models of care in ED and included 14 studies (10 observational studies, one randomised trial reported in two papers, and two reviews) [23]. The authors suggested that nurse practitioners were cost-effective and had a positive impact on waiting times and patient satisfaction, however the impact of nursing models of care on ED services (e.g. ED re-presentation rates) remains unclear. Our review included only one trial [39] that evaluated nurse practitioners managing musculoskeletal conditions in ED and reinforces the authors suggestion that there is a need for further high-quality research evaluating the effectiveness of nurse practitioners delivering musculoskeletal care in ED [23]. While the findings of our review expand on previous research it also highlights knowledge gaps in this field of research.

### Strengths and limitations

To our knowledge, this is the first systematic review and meta-analysis to include only randomised trial evidence on the effectiveness of nurse practitioners and allied health clinicians managing musculoskeletal conditions in ED. Our review provides certainty of evidence (GRADE assessment) for primary and secondary outcomes. The extensive search strategy was employed to identify relevant trials evaluating both patient (e.g. pain intensity, disability) and health service outcomes (e.g. patient-flow). Additionally, there were no restrictions applied to language in our review, and we included nursing and allied health models of care. While our search identified other allied health models of care (i.e. chiropractic and occupational therapy) for the management of musculoskeletal conditions in ED, these studies did not meet the eligibility criteria (i.e. did not compare allied health care to usual ED physician care) and were therefore excluded. We were able to analyse data to describe the effectiveness of physiotherapy care on pain and disability outcomes for patients presenting with musculoskeletal conditions.

There are some limitations to our review. Firstly, the pooled estimates were analysed using data provided in two to three published trials of varying risk of bias (i.e. low and high risk of bias) and sample sizes (i.e. 45 to 110 participants). Additionally, heterogeneity must be considered when interpreting the review findings. The meta-analyses in this review included few studies and hence it was inappropriate to report the prediction interval for hetergeneity [43]. Instead, between-study variance in effect sizes was evaluated using the tau-statistic and reported in conjunction with the I^2^ statistic to describe variance in sampling error that was due to variation in true effects [43]. Furthermore, due to the small number of included trials it was not possible to conduct subgroup analyses to explore differences between nurse practitioners and allied health clinicians such as physiotherapists. Finally, the findings of this review only provide outcomes at immediate, short and intermediate-term. No trials provide data at long-term follow-up (greater than 12-months).

There is limited data on the impact of nurse practitioners and allied health clinicians managing musculoskeletal conditions on inpatient admission, ED re-presentation rates, and adverse events. Current data on patient-flow outcomes (i.e. length of stay, wait and treatment-times) come from observational studies and are at inherent risk of selection bias. The evidence presented in this review comes from randomised controlled trials and highlights that uncertainty exists toward the effectiveness of physiotherapy and nurse practitioners managing musculoskeletal conditions in ED. Our review identified only two trials that provided data on inpatient admission and ED re-presentation rates, and reported on adverse events, and one of these trials was limited due to small sample size (n = 78) and poor follow-up (21% of participants lost to follow-up) [17, 38]. Additionally, one of these trials did not report if the patient re-presented to ED for the same condition or a new complaint and this must be considered when interpreting the data [36].

## Conclusion

While previous observational studies [10, 13, 15, 21] have explored nurse practitioners and physiotherapists influence on wait times and ED length of stay, there is a need for randomised evidence. Our review found very-low and low-quality evidence of no effect of allied health and nursing models of care for the management of musculoskeletal conditions in ED. Future trials should investigate the impact of nurse practitioners and allied health models of care for managing musculoskeletal conditions on health service outcomes such as patient-flow, re-presentation rates and adverse events, compared to usual medical care.

### Electronic supplementary material

Below is the link to the electronic supplementary material.

Supplementary Material 1

Supplementary Material 2

## Data Availability

All data generated during this study are included in this published article [and its supplementary information files].
