# Supplementary material for: The effectiveness of allied health and nurse practitioner models-of-care in managing musculoskeletal conditions in the emergency department: a systematic review and meta-analysis
Source: BMC Emerg Med. 2024 Jan 17;24:13. doi: 10.1186/s12873-023-00925-4 (PMC10795385; doi:10.1186/s12873-023-00925-4)
Supplement: Supplementary file 1 — Supplementary Material 1 [file 12873_2023_925_MOESM1_ESM.docx]

**Appendix 1.** Database search string for EMBASE and MEDLINE

EMBASE via Ovid

MEDLINE via Ovid

1 emergency medical service*.mp.
2 triage.mp.
3 urgent care.mp.
4 emergency.mp.
5 casualty.mp.
6 "accident and emergency".mp.
7 trauma centre.mp.
8 emergency department.mp.
9 emergency setting.mp.
10 pre-hospital.mp.
11 1 or 2 or 3 or 4 or 5 or 6 or 7 or 8 or 9 or 10
12 physiotherap*.mp.
13 physical therap*.mp.
14 exercise therap*.mp.
15 kinesiotherap*.mp.
16 exercise physiolog*.mp.
17 manual therap*.mp.
18 chiropractic.mp.
19 exercise.mp.
20 nurse.mp.
21 nursing*.mp.
22 emergency nurse.mp.
23 health personnel.mp.
24 12 or 13 or 14 or 15 or 16 or 17 or 18 or 19 or 20 or 21 or 22 or 23
25 musculoskeletal pain.mp.
26 musculoskeletal disease.mp.
27 low back pain*.mp.
28 25 or 26 or 27
29 11 and 24 and 28
