# Supplementary material for: The effectiveness of allied health and nurse practitioner models-of-care in managing musculoskeletal conditions in the emergency department: a systematic review and meta-analysis
Source: BMC Emerg Med. 2024 Jan 17;24:13. doi: 10.1186/s12873-023-00925-4 (PMC10795385; doi:10.1186/s12873-023-00925-4)
Supplement: Supplementary file 2 — Supplementary Material 2 [file 12873_2023_925_MOESM2_ESM.docx]

**Appendix 2.** GRADE Framework

- Risk of bias: downgrade by one level if most information is from studies at high risk of bias (i.e. PEDro score <7).1
- Inconsistency: for pooled analyses downgrade by one level if heterogeneity was large (I2 statistic value >50%, representing potentially substantial heterogeneity).2 Tau^2^ statistic was used to assist in describing heterogeneity of the study results (i.e. between-study variance).^3^
- Indirectness: We did not assess indirectness as patients, interventions and comparators were similar across studies.^4^
- Imprecision: Continuous outcomes: downgrade by one level if the limits of the 95% confidence interval are excessively wide (i.e. 20 points different to the point estimate; twice the minimal clinically important difference of 10 points on a 100-point scale), or sample size was less than 400.^4,5^
- Small study effects: downgrade by one level if more than 25% of participants were from small studies (< 100 participants per arm).^6^

REFERENCES

1. Guyatt GH, Oxman AD, Vist G, et al. GRADE guidelines: 4. Rating the quality of evidence - study limitations (risk of bias). *J Clin Epidemiol.* 2011;64(4):407-415.
2. Guyatt GH, Oxman AD, Kunz R, et al. GRADE guidelines: 7. Rating the quality of evidence- inconsistency. *J Clin Epidemiol.* 2011;64(12):1294-1302.
3. Borenstein M. Research Note: In a meta-analysis, the I2 index does not tell us how much the effect size varies across studies. Journal of Physiotherapy. 2020;66(2):135-9.
4. Guyatt GH, Oxman AD, Kunz R, et al. GRADE guidelines: 8. Rating the quality of evidence- indirectness. *J Clin Epidemiol.* 2011;64(12):1303-1310.
5. Guyatt GH, Oxman AD, Kunz R, et al. GRADE guidelines 6. Rating the quality of evidence- imprecision. *J Clin Epidemiol.* 2011;64(12):1283-1293.

Guyatt GH, Oxman AD, Montori V, et al. GRADE guidelines: 5. Rating the quality of evidence- publication bias. *J Clin Epidemiol.* 2011;64(12):1277-1282
